# A Patient with Eosinophilic Esophagitis and Herpes Simplex Esophagitis: A Case Report and Literature Review

**DOI:** 10.1155/2021/5519635

**Published:** 2021-05-25

**Authors:** Hemnishil K. Marella, Jiten P. Kothadia, Nasir Saleem, Bilal Ali, Yousef Abdel-Aziz, Vamsee Mupparaju, Twisha Oza, Abdallah Azouz, Colin W. Howden

**Affiliations:** ^1^Division of Gastroenterology and Hepatology, University of Tennessee Health Science Center, Memphis, TN 38104, USA; ^2^Methodist University Hospital, Memphis, TN 38104, USA; ^3^Department of Pathology, University of Tennessee Health Science Center, Memphis, TN 38104, USA

## Abstract

Acute herpes simplex esophagitis (HSE) is common in immunocompromised patients. Eosinophilic esophagitis (EoE) is characterized by immune-mediated eosinophil-predominant esophageal inflammation. We report a patient with human immunodeficiency virus infection who presented with dysphagia and odynophagia and was found to have HSE and EoE. The combination of these two relatively rare conditions suggests possible predisposition.

## 1. Introduction

Eosinophilic esophagitis (EoE) is a chronic immune and antigen-mediated disorder characterized by an eosinophil-predominant inflammation of the esophagus resulting in esophageal dysfunction symptoms [1]. Although once considered rare, its reported incidence is increasing partly due to better recognition and improved understanding of the disease process [2–4]. Its most frequent symptoms in adults are dysphagia and food impaction [1]. The diagnosis of EoE is established on clinical, endoscopic, and histologic grounds after other etiologies of symptoms and esophageal eosinophilia are excluded. Histological diagnosis is based on the presence of at least 15 eosinophils per high-power field (hpf) on esophageal mucosal biopsies [1]. Environmental factors, atopy, genetics, and impaired esophageal epithelial barrier function are possible predisposing factors for EoE [1].

Herpes simplex esophagitis (HSE) is an acute viral infection of the esophagus that may cause odynophagia and/or dysphagia, chest pain, and fever [5]. Herpes simplex virus (HSV) lesions are typically found in the mid-to-distal esophagus and can be confirmed histologically by immunohistochemistry, viral culture, or polymerase chain reaction [6]. The occurrence of concomitant HSE and EoE is rare [7–10]. We report a case of concomitant HSE and EoE in an immunocompromised patient and have described a well-referenced review on the subject.

## 2. Case Report

A 51-year-old African American woman with a past medical history significant for a history of gastroesophageal reflux disease and HIV presented with a one-year history of dysphagia and odynophagia. She was receiving elvitegravir, cobicistat, emtricitabine, and tenofovir alafenamide for HIV infection. Her most recent CD4 count was 207 cells/mm^3^, and her viral load was 84,700 copies/mL. She complained of odynophagia and dysphagia at the level of her neck, as well as some epigastric discomfort. She reported weight loss of 5 pounds over six months. She denied any history of atopic disorders. She noticed painful lesions at the edge of her tongue. She had been taking omeprazole 40 mg daily for the preceding six months without symptom improvement. Over the preceding 2-3 years, her CD4 count had been ≤200 with high viral loads. Despite previous poor compliance, she had recently been compliant with all her HIV medications.

**Figure 1:**
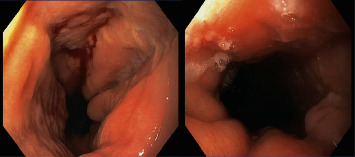
Multiple, discrete, 1-2 mm shallow ulcers with heaped-up edges in the distal esophagus.

**Figure 2:**
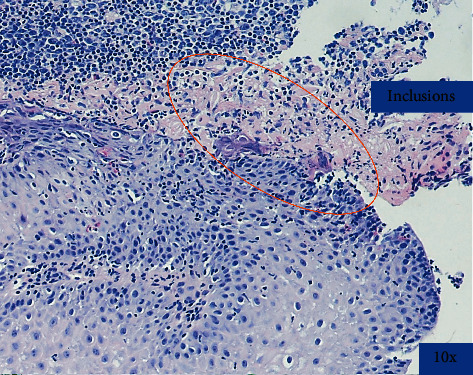
Margination, multinucleation, and moulding consistent with HSV infection.

**Figure 3:**
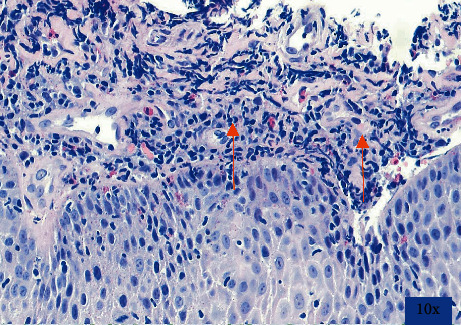
Presence of eosinophils (20/hpf), eosinophilic microabscesses, and eosinophil degranulation and Bazel zone hypertrophy.

On examination, there were several 2 mm shallow ulcers at the edge of her tongue but no evidence of oral *Candida* infection. Her blood count, biochemistry, and liver function tests were within normal limits. Before EGD, she was started on empiric fluconazole because of suspicion for esophageal candidiasis. At EGD, there were multiple, discrete, 1-2 mm shallow ulcers with heaped-up edges in the distal esophagus (Figure 1). The stomach and duodenum were unremarkable. Proximal, mid, and distal esophageal biopsies revealed margination, multinucleation, and moulding consistent with HSE (Figure 2). Additionally, there were 20 eosinophils/hpf (Figure 3) along with eosinophil microabscesses, extracellular eosinophil granules, and Bazel zone hypertrophy. Immunohistochemical staining was positive for HSV. Grocott methenamine silver and periodic acid-Schiff stains did not show fungal microorganisms. The PCR test for cytomegalovirus (CMV) and Epstein–Barr virus (EBV) was negative. The patient was treated with acyclovir 400 mg five times daily for 14 days and was continued omeprazole 40 mg daily. Following treatment, the patient reported significant improvement in her dysphagia and odynophagia.

## 3. Discussion

The clinical separation between EoE and GERD is challenging, and an overlap in symptom presentation is recognized. In our case, the major clinical symptom of dysphagia, the histological findings (20 eosinophils/hpf), and the lack of resolution of the symptoms despite the patient taking omeprazole in the preceding months are the main distinguished factors that point towards EoE rather than GERD. The occurrence of concomitant HSE and EoE is rare [7–10]. Our patient's case further adds to the evidence base for a possible relationship between EoE and HSE. It is unknown whether our patient had EoE or HSE first. It is unlikely that these two conditions would coexist by chance alone. With these observations in mind, it is essential to consider possible mechanisms for the coexistence.

First, HSE could predispose to EoE. HSE damages the esophageal mucosa resulting in immune hyperactivity and initiation of the inflammatory cycle [11]. Increased esophageal permeability from mucosal damage may allow access to antigenic stimuli from ingested food and environmental antigens. Antigen-presenting cells can induce T helper 2 (Th2) cells leading to eosinophilic infiltration/inflammation of the esophageal lamina propria and submucosa [11]. Also, acute inflammation resulting from HSE can induce predominantly neutrophilic infiltrate, and EoE may be masked if there is an underlying disease [12].

Alternatively, EoE might predispose patients to HSE. Interleukin 13 (IL-13) has been implicated in the pathogenesis of EoE [11]. IL-13 leads to downregulation of the desmosomal protein, desmoglein-1 [11, 13]. Downregulation of intracellular junction proteins can lead to exacerbation of inflammation and may also lead to impairment of the mucosal barrier, thereby facilitating candidiasis, HSV, and other viral infections [11, 13].

On review of the literature, we found eight adult cases related to EoE and HSE reported (Table 1). The average age was 24.8 years, and three had a definite history of atopy. HSE preceded EoE in three out of eight cases. EoE preceded HSE in three out of the eight cases. Concomitant HSE and EoE were detected in three cases, including our case. Patients with atopy were diagnosed with HSE before EoE. However, it is imperative to ask all patients with EoE about atopic disorders and food and environmental allergies. In most of the previously reported cases, treatment comprised a proton-pump inhibitor and acyclovir/valacyclovir (Table 1).

Additionally, the association between EoE and HIV is not well established. There is only one case report in the literature about the occurrence of EoE in HIV patients [14]. A recently published cross-sectional study showed that HIV patients are twice as likely to have EoE compared to those without HIV [15]. The proposed mechanism of association between EoE and HIV is due to Th2 inflammation mediated by the cytokine thymic stromal lymphopoietin (TSLP), and there are elevated levels of TSLP in patients with HIV [15].

In conclusion, regardless of a patient's immune status, HSE and EoE can occur simultaneously or successively. However, it is challenging to identify the causal relationship between EoE and HSE, and one disease may predispose to the other. Future studies are needed to evaluate the pathogenicity and coexistence of these two disease processes.

## Figures and Tables

**Table 1:** Summary of clinical characteristics of published cases associated with eosinophilic esophagitis and herpes simplex virus esophagitis.

Author (year)	Country	Age of the patient (yr)	Sex	Immunocompromised (yes/no)	Atopy	HSV or EoE diagnosed first (or concurrently)	Reported treatment
Monsanto et al. [9]	Portugal	20	M	No	Asthma	HSV 6 weeks before	Acyclovir + PPI followed by fluticasone
Machicado et al. [8]	USA	18	M	No	Allergic rhinitis and wheat allergy	Concurrently	Acyclovir (3 days) + valacyclovir (11 days) + PPI
Zimmermann et al. [10]	Switzerland	27	F	No	Unknown	EoE 5 years before	Acyclovir + PPI followed by fluticasone
		25	F	No	Unknown	EoE	Acyclovir
		30	M	No	Unknown	EoE 6 years before	Acyclovir
		28	M	No	Asthma and allergic rhinitis	HSV 9 weeks before	None (asymptomatic and lost to follow-up)
		29	M	No	Unknown	Concurrently	Acyclovir + PPI
Iriarte Rodríguez et al. [7]	Spain	21	M	No	None	HSV 12 weeks before	Acyclovir + lidocaine

## Data Availability

The data used to support the findings of this study are available from the corresponding author upon request.
